# Granulomatous Myositis Associated with Myasthenia Gravis: A Rare Case

**DOI:** 10.7759/cureus.5090

**Published:** 2019-07-07

**Authors:** Shumaila M Iqbal, Linda Burns, Cassandra Zhi

**Affiliations:** 1 Internal Medicine, University at Buffalo / Sisters of Charity Hospital, Buffalo, USA; 2 Rheumatology, Buffalo Rheumatology, Buffalo, USA; 3 Internal Medicine, Drexel University College of Medicine, Philadelphia, USA

**Keywords:** myasthenia gravis, granulomatous myositis, striational antibody

## Abstract

Myasthenia gravis (MG) is an immune-mediated disease of the neuromuscular junction mediated by anti-acetylcholine receptor (AChR) antibodies (Ab). Granulomatous Myositis (GrM) is a histological diagnosis characterized by the presence of epithelioid granuloma in striated muscles. Few cases describing the presence of concomitant thymoma and non-thymoma-related MG with GrM have been reported. This present case is an addition to the literature describing the presence of concomitant thymoma and non-thymoma-related MG with GrM. The patient described is a 77-year-old male who started developing weakness and atrophy involving the musculature of the bilateral lower and upper extremities. Initial laboratory workup showed an elevated level of serum creatine phosphokinase (CPK) of 1,231 U/ L (reference range: 22 to 198 U/L). The right quadriceps muscle biopsy performed showed inflammatory infiltrates containing eosinophils, plasma cells, and lymphocytes forming multinucleate giant cells consistent with a diagnosis of GrM. Detailed laboratory and imaging work conducted to rule out an underlying cause of GrM showed elevated titers of AChR Ab (79.50 nmol/L, reference range: <0.02 nmol/L) and striational Ab (titer: 1:320, reference range < 1:120). A positive repetitive nerve stimulation test for the left ulnar nerve (decrement in the amplitude of muscle action potential by 13%) further confirmed the diagnosis of MG concomitant with GrM. Computed tomography of the chest was negative for the presence of a thymoma. The patient was started on treatment with oral prednisone and mycophenolate mofetil, which resulted in an improvement of symptoms and the downward trending of serum CPK level.

## Introduction

Myasthenia gravis (MG) is an immune-mediated disease of the neuromuscular junction, predominantly affecting the bulbar muscles. This disease is mediated by the anti-acetylcholine receptor (ACh) antibody (Ab) or rarely by muscle-specific tyrosine kinase (MuSK) Ab, lipoprotein-related protein 4 (LRP4), or agrin [1-2]. The simultaneous presence of striational Ab is usually associated with late-onset, severe, or thymoma-related MG [3-4]. These striational Abs react with epitopes on muscle proteins and bind in a cross-striational pattern to skeletal and heart muscle tissue sections [1,5]. Three major targets for striational Ab are the calcium release channel of the sarcoplasmic reticulum known as the ryanodine receptor (Ryr), titin, a gigantic filamentous muscle protein essential for muscle structure, function, and development; and the alpha subunit of the muscular voltage-gated potassium channel Kv 1.4 [5-6].

Granulomatous myositis (GrM) is a histological diagnosis characterized by the presence of epithelioid granuloma in striated muscles. GrM is most frequently associated with sarcoidosis [7-8]. The other less common causes include several infectious diseases, foreign-body giant-cell reaction, inflammatory bowel diseases, malignancy (lymphoma), and myasthenia gravis [9-10]. The thymoma-associated subtype of MG is the most common one reported with GrM [1,3]. Our case is unique, as it reports an intriguing case of non-thymoma related MG with GrM.

## Case presentation

Our patient was a 77-year-old male with a past medical history significant for type-2 diabetes mellitus, dyslipidemia, and hypertension. He started developing weakness and atrophy, initially involving the musculature of the lower extremities followed by the upper extremities. He also reported a significant weight loss of 40 pounds from his baseline weight. The physical examination demonstrated a significant loss of muscle mass in all muscle groups of the upper and lower extremities. This patient did not exhibit symptoms involving bulbar muscles. The Medical Research Council Manual Muscle Testing Scale was utilized to grade muscle strength for different muscle groups, as listed: neck flexion 4, neck extension 5, arm abduction, extension, and flexion 4, elbow extension and flexion 4, wrist extension and flexion 3, fingers’ extension, flexion, and abduction 3, hip extension 4, hip flexion and abduction 3, knee extension and flexion 4, ankle dorsiflexion and plantarflexion 4. Muscle tone remained normal without any restricted range of motion. Deep tendon reflexes were decreased all over, with a value of 1+/5. Sensory perception was intact on both sides of the body. Initial laboratory workup showed an elevated creatine phosphokinase (CPK): 1,231 U/ L (reference range: 22 to 198 U/L) and myoglobin: 1,787 ng/ml (reference range: 0 to 85 ng/Ml). A detailed autoimmune workup performed showed negative serologic markers of antinuclear Ab, anti-Smith Ab, anti-U1 ribonucleoprotein Ab, anti-Jo-1 Ab, anti-Sjögren's-syndrome-related antigen A Ab, anti-Sjögren's-syndrome-related antigen B Ab, myeloperoxidase-anti-neutrophil cytoplasmic antibody, and proteinase anti-neutrophil cytoplasmic antibody. Thyroid stimulating hormone levels were within the normal range (2.3 mIU/L, reference range: 0.4-4.0 mIU/L).

**Figure 1:**
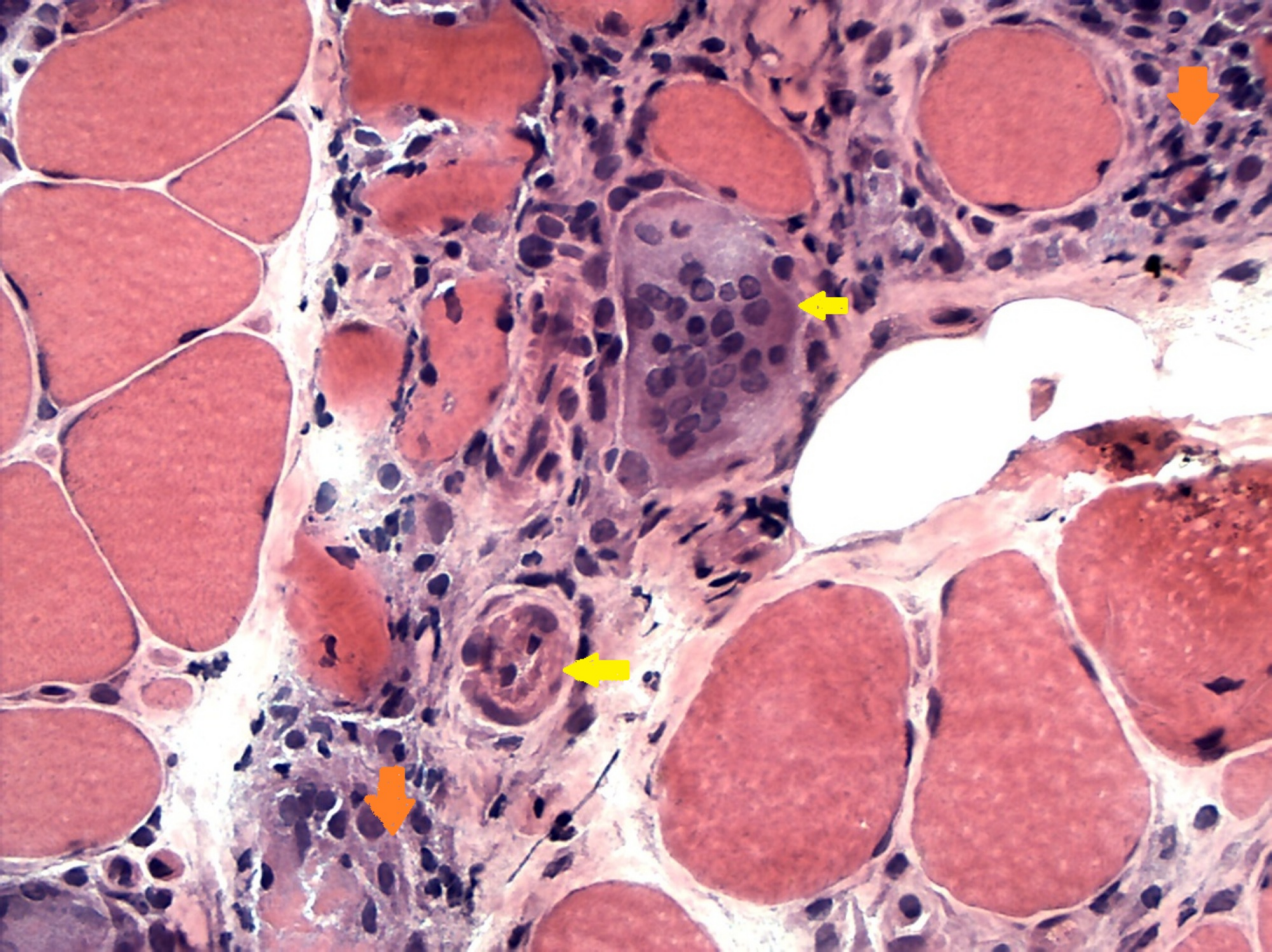
Frozen hematoxylin and eosin staining showing marked necrosis and inflammatory cell infiltrate of the muscle with extension to the fascia consisting of lymphocytes, plasma cells, eosinophils, macrophages, and multinucleated giant cells Orange-colored arrowhead pointing towards the inflammatory infiltrate; yellow-colored arrowhead pointing towards multinucleated giant cells.

A right quadriceps muscle biopsy was performed, which demonstrated necrosis and atrophy of muscle fibers and inflammatory infiltrate of eosinophils, plasma cells, and lymphocytes forming multinucleated giant cells consistent with granulomatous myositis (GrM) (Figure 1).

This was followed by detailed laboratory and imaging studies to rule out the underlying cause for this GrM, which showed positive testing for elevated titers of AChR Ab (79.50 nmol/L, reference range: <0.02 nmol/L) and striational Ab (titer: 1:320, reference range < 1:120). Computed tomography of the chest did not show the presence of a thymoma. Repetitive nerve stimulation testing for the left ulnar nerve showed a decrement in the amplitude of muscle action potential by 13%, further confirming the diagnosis of non-thymoma-related MG concomitant with GrM. Other causes of GrM were also ruled out. For example, sarcoidosis was ruled out due to the patient’s normal serum levels of angiotensin-converting enzyme and 25-hydroxycholecalciferol levels with no mediastinal or cervical lymphadenopathy evident on chest imaging. Testing was negative for infection with syphilis and human immunodeficiency virus, which ruled out infection as a cause of myositis. Occult malignancy was also ruled out by a negative positron emission tomography scan for increased tracer uptake throughout the body.

The patient was started on treatment with 60 mg of oral prednisone for MG-associated GrM. We were successful in tapering down the dose of prednisone to 5 mg daily with the addition of mycophenolate mofetil 1000 mg twice a day as steroid-sparing medication. Therapy resulted in a moderate improvement in symptoms along with a descending trend in the levels of CPK and myoglobin.

## Discussion

This is an unusual case of histologically proven GrM, with non-thymoma-related MG. Our patient’s serum was positive for striated muscle Ab and AChR Ab. Striational Ab may or may not be found in patients with MG. If present, the antibody targets epitopes on muscle proteins, including titin, ryanodine receptor, and Kv 1.4 [5-6]. The pattern of coupling with the antigens was observed to be cross-striational. Its presence is usually indicative of severe disease in all subgroups of myasthenia gravis [5] and can, therefore, be used as a prognostic determinant in patients with MG [11].

Myasthenia gravis tends to be more severe if a concomitant thymoma is also present. Striational Ab is also more frequently discovered in this subcategory of MG as compared to the other categories [12-13]. An absence of striational Ab can strongly exclude thymoma in patients with MG [14]. However, its presence has also been reported in patients with MG without thymoma as well [11-15]. This was the case seen in our patient. The patient’s serum remained positive for high titers of striational Ab but CT chest did not reveal any presence of a thymoma.

Histologically, the myocardial and skeletal muscle tissues evaluated in patients with MG presenting with striational Ab revealed inflammatory infiltrates containing multinucleated giant cells and lymphocytes, which were mainly CD8+ and CD68+ and, rarely, CD4+ [16-17]. Our patient had undergone a skeletal muscle biopsy of the quadriceps muscle, which only reported GrM with marked necrosis and inflammatory infiltrates extending to the fascial sheath, which contained plasma cells, lymphocytes, and eosinophils forming multinucleated giant cells.

Treatment for acute GrM requires a combination of corticosteroids, plasmapheresis, and intravenous (IV) immunoglobulins due to immune and inflammatory cell-mediated muscle damage [18]. Our patient’s symptoms had also shown moderate improvement with oral corticosteroids. We also added mycophenolate mofetil 1000 mg twice a day, along with a gradual tapering of the steroid dose for maximum treatment effect while minimizing the long-term complications of treatment therapy with steroids.

## Conclusions

The coexistence of MG with GrM has rarely been reported. Patients with MG who are also seropositive for striational Ab should be evaluated for inflammatory myopathies, as this antibody is found to be highly prevalent in patients with GrM. Striational Ab targets the contractile filaments of muscle fibers, which might also be the causative factor of inflammatory myositis. Nevertheless, the exact pathophysiologic mechanism of this phenomenon is yet to be determined.
